# The Periodicals

**Published:** 1872-06

**Authors:** 


THE PERIODICLES.—The *Atlantic Monthly* for July is replete with many interesting articles.

The departments of literature and science are each filled with vivid and pleasant discussions concerning the literature of the times and some of the recent discussions in science which have engaged the minds of scientific circles. Dr. O. W. Holmes gives part VII of his entertaining articles on the Poet of the breakfast-table. Mr. John A. Bolles, naval solicitor commences in this number an explanation of the reasons why Semmes of the Alabama was not tried.

We have missed the *Atlantic* from our table for some time and are glad to welcome back its smiling countenance.

